# Draft Genome Sequence of Candida auris Strain LOM, a Human Clinical Isolate from Greater Metropolitan Houston, Texas

**DOI:** 10.1128/MRA.00532-19

**Published:** 2019-06-20

**Authors:** S. Wesley Long, Randall J. Olsen, Hoang A. T. Nguyen, Matthew Ojeda Saavedra, James M. Musser

**Affiliations:** a Center for Molecular and Translational Human Infectious Diseases Research, Department of Pathology and Genomic Medicine, Houston Methodist Research Institute and Houston Methodist Hospital, Houston, Texas, USA; b Department of Pathology and Laboratory Medicine, Weill Cornell Medical College, New York, New York, USA; Broad Institute

## Abstract

Candida auris is an emerging pathogen of considerable public health importance. We present the draft genome sequence of a strain recently cultured from the urine of a patient hospitalized in the greater Houston metropolitan region. Two combined Oxford Nanopore sequencing runs provided sufficient data to rapidly generate a draft genome.

## ANNOUNCEMENT

Candida auris recently has emerged worldwide and caused outbreaks in health care facilities (1, 2). An isolate of C. auris was cultured from the urine of a patient from the Houston metropolitan region on 5% sheep blood tryptic soy agar and identified by Brucker matrix-assisted laser desorption ionization–time of flight (MALDI-TOF) analysis using the research use only (RUO) v4.1.80 database. The patient had not recently traveled outside the United States and had transferred from a long-term-care facility. This work was approved by our institutional review board (IRB1010-0199).

The organism was classified as present on admission. Given the potential infection control and public health importance of this isolate, we rapidly characterized its genome using two Oxford Nanopore GridION runs using FLO-MIN106 flow cells and the Guppy v2.0.10 base caller. DNA was extracted from overnight growth on solid agar using ballistic lysis with FastPrep matrix B (first run) or Y (second run) and the Qiagen DNA blood and tissue kit. The first GridION run used the rapid barcoding kit (catalog number SQK-RBK004), yielding 1.29 million reads and 3.48 gigabases (Gb) of sequence with an average read length of 2.7 kb. The second run used the ligation sequencing kit (catalog number SQK-LSK109) with a long-read wash, yielding 2.98 million reads and 13.72 Gb of sequence with an average read length of 4.6 kb. The reads from both runs were combined and filtered using Filtlong v0.1.1 with a 5-kb-read cutoff and 100-fold coverage (https://github.com/rrwick/Filtlong). Unicycler v0.4.3 was used to assemble the genome with miniasm and pilon polishing (3). The assembly had 9 contigs (total length, 12,293,266 bp). The largest contig was 4,297,164 bp, and the *N*_50_ value was 2,304,466 bp. The average GC content was 45.19%.

**FIG 1:**
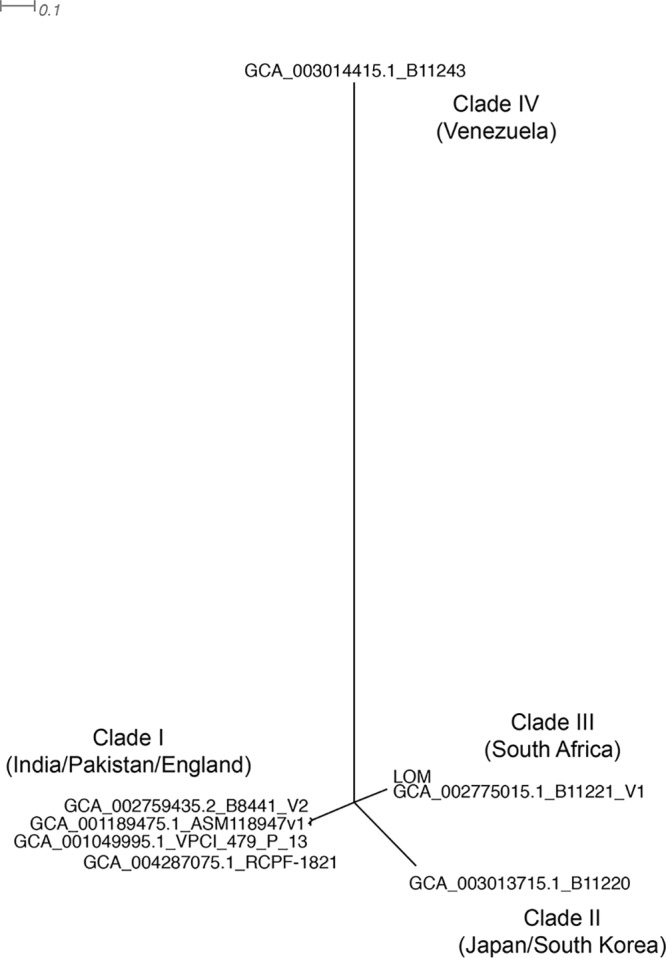
Neighbor-joining radial phylogenetic tree showing the relationship of C. auris strain LOM to the 7 reference strain genomes, B11221 (GenBank accession number GCA_002775015), RCPF-1821 (GCA_004287075), 6684 (GCA_001189475), B8441 (GCA_002759435), B11220 (GCA_003013715), B11243 (GCA_003014415), and VPCI 479/P/13 (GCA_001049995). The four clades and their respective geographic associations are indicated.

We compared our assembly with 7 reference genome assemblies in GenBank (strains B11221, RCPF-1821, 6684, B8441, B11220, B11243, and VPCI 479/P/13) using progressiveMauve v2.4.0 and discovered that our 7 longest contigs correspond to the 7 chromosomes present in other C. auris strains (4). Phylogenetic analysis using MUMmer v4.0 (with the show-snps setting) showed that C. auris strain LOM is most closely related to strain B11221, a strain from clade III (1,177 single-nucleotide polymorphisms [SNPs] distant) (5, 6). Prephix and phrecon (https://github.com/codinghedgehog/) were used to generate an alignment from the MUMmer SNPs to create a neighbor-joining tree using FastTree2 (Fig. 1) (7). The LOM genome has the *erg11* gene with an F126L amino acid replacement that is common to clade III strains (6).

This work further demonstrates the usefulness of real-time long-read whole-genome sequencing to rapidly provide relevant information concerning emerging pathogens of significant concern (8, 9). The availability of this high-quality draft genome sequence will serve as a useful resource if additional isolates are recovered in the greater Houston metropolitan area. The sequence data also will assist translational research efforts designed to more fully understand the molecular mechanisms underlying host interactions in an emerging pathogen that has substantial detrimental public health potential.

### Data availability.

The BioProject accession number for C. auris strain LOM is PRJNA540998, the GridION runs are in the SRA (numbers SRR9017243 and SRR9017244), and the draft genome is in GenBank (accession number SZYF00000000). The 7 reference strains and their GenBank genome assembly accession numbers are as follows: B11221 (GCA_002775015), RCPF-1821 (GCA_004287075), 6684 (GCA_001189475), B8441 (GCA_002759435), B11220 (GCA_003013715), B11243 (GCA_003014415), and VPCI 479/P/13 (GCA_001049995).
